# Maternal exposure to heat and its association with miscarriage in rural KwaZulu-Natal, South Africa: A population-based cohort study

**DOI:** 10.1177/17455057241259171

**Published:** 2024-07-26

**Authors:** Yoshan Moodley, Kwabena Asare, Frank Tanser, Andrew Tomita

**Affiliations:** 1 Africa Health Research Institute, KwaZulu-Natal, South Africa; 2 Faculty of Health and Environmental Sciences, Central University of Technology, Bloemfontein, South Africa; 3 Division of Health Systems and Public Health, Faculty of Medicine and Health Sciences, Stellenbosch University, Cape Town, South Africa; 4 School of Nursing and Public Health, University of KwaZulu-Natal, Durban, South Africa; 5 Centre for the AIDS Programme of Research in South Africa, KwaZulu-Natal, South Africa; 6 Health Economics and HIV and AIDS Research Division (HEARD), University of KwaZulu-Natal, Durban, South Africa; 7 Centre for Epidemic Response and Innovation, School for Data Science and Computational Thinking, Stellenbosch University, Stellenbosch, South Africa; 8 South African Centre for Epidemiological Modelling and Analysis (SACEMA), Stellenbosch University, South Africa; 9 Centre for Rural Health, University of KwaZulu-Natal, Durban, South Africa; 10 KwaZulu-Natal Research Innovation and Sequencing Platform, University of KwaZulu-Natal, Congella, South Africa

**Keywords:** climate change, hot temperature, miscarriage, sub-Saharan Africa

## Abstract

**Background:**

We sought to improve the current understanding of how climate change impacts women’s reproductive health in sub-Saharan Africa.

**Objectives:**

We investigated the relationship between maternal heat exposure and miscarriage (pregnancy ending before 20 weeks gestation) in a South African setting.

**Design:**

Population-based cohort study.

**Methods:**

Our study involved data for pregnancies collected via a health and demographic surveillance system in rural KwaZulu-Natal, South Africa between 2012 and 2016. Data from the South African Weather Service were used to compute maternal exposure to heat during the following time windows for each pregnancy: during the month preceding conception (T1) and during the week preceding the study outcome (either a miscarriage or no miscarriage, T2). Heat exposure was operationalized as a continuous variable and defined as the number of days that a mother was exposed to a mean daily temperature of > 26.6°C (A “hot day,” equivalent to a mean daily temperature of > 80°F) during T1 or T2. Binary logistic regression was used to investigate the relationship between maternal heat exposure and miscarriage.

**Results:**

A total of 105/3477 pregnancies included in our analysis ended in miscarriage (3.0%). Each additional hot day during T1 was associated with a 26% higher odds of miscarriage (odds ratio: 1.26; 95% confidence interval: 1.15–1.38). No significant associations were observed between maternal heat exposure during T2 and the odds of miscarriage (odds ratio: 0.94, 95% confidence interval: 0.73–1.20). The relationship between maternal heat exposure during T1 and the odds of miscarriage was J-shaped.

**Conclusion:**

There is a clear relationship between maternal heat exposure during the month preceding conception and miscarriage in our sub-Saharan African setting. Given the lack of feasible strategies to reduce pregnancy loss associated with prevailing high temperatures in sub-Saharan Africa, progressive climate change will likely exacerbate existing challenges for women’s reproductive health in this region.

## Introduction

Climate change has accelerated the rise in global temperatures first observed during the 1960s.^1^ Sub-Saharan Africa is particularly vulnerable to the hazardous effects of climate change, which has already exacerbated existing high ambient temperatures in the region.^2^ In addition, sub-Saharan Africa is constantly plagued by resource constraints that represent a barrier to the effective roll-out of climate change adaptation or mitigation strategies.^3,4^

In South Africa, increasing temperatures have dire implications for the health of socially vulnerable populations who are currently impacted by a quadruple burden of disease.^5,6^ The association between high ambient temperatures and an increased risk of adverse health outcomes (including mortality, heat stress, and infectious diseases) is well established in the South African context^7^ and is congruent with the published literature emanating from industrialized regions of the world.^8–10^ A recent systematic review by Chersich et al.,^11^ of studies conducted outside the African continent, suggests that maternal exposure to increasing temperatures during pregnancy is associated with a higher odds of preterm birth or stillbirth. There have been several studies published since then which continue to demonstrate a harmful association between higher ambient temperature exposure and adverse pregnancy outcomes. A study from Fuzhou, China reported a 10% increase in adverse pregnancy outcomes for each 1°C increase in mean temperature exposure during early pregnancy.^12^ Similarly, a population-based study by Ren et al.^13^ found an association between heat exposure during pregnancy and a higher risk of preterm birth in China. In Iran, a 1°C increase in daily temperature exposure during pregnancy was found to be associated with a 3%–15% higher adjusted risk of preterm birth.^14^ In their analysis of birth outcomes data from Western Australia, Nyadanu et al.^15^ reported that there was a 19% higher risk of stillbirth in women who experienced acute heat stress during the 6 days prior to the pregnancy outcome. Furthermore, a population-based study from Taiwan found that the risk of stillbirth was higher in the context of extreme heat exposure, particularly when this exposure occurred during the third trimester of pregnancy.^16^ Although most of the evidence around the impact of higher temperatures on pregnancy outcomes is based on studies of the prenatal period, maternal exposure to high temperatures during the preconception period is also important. From a biological standpoint, high temperatures during the preconception period can interfere with gametogenesis, which in turn has consequences for fetal development and subsequent fetal outcomes.^11,17^ With excessively high poverty, human immunodeficiency virus (HIV) infection, and maternal mortality rates,^18–20^ sub-Saharan African women of childbearing age are a prime example of an already vulnerable population which could potentially suffer additional challenges related to their reproductive health due to increasing temperatures resulting from climate change.

We sought to improve the current understanding of how climate change impacts women’s reproductive health in sub-Saharan Africa using one of the region’s largest population-based datasets from rural KwaZulu-Natal Province, South Africa to investigate the impact of maternal heat exposure and miscarriage.

## Methods

### Study design and setting

**Figure 1.**
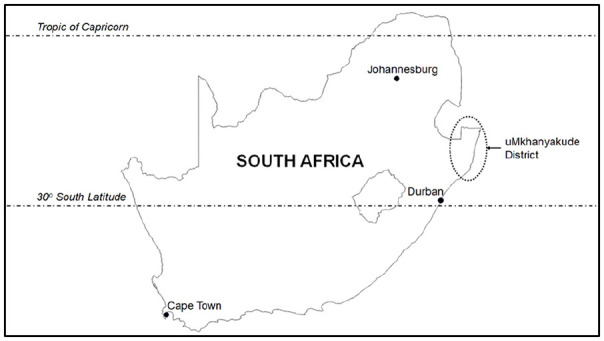
Location of the uMkhanyakude District, South Africa.

This was a population-based cohort study involving pregnant women from a rural community in the uMkhanyakude District of KwaZulu-Natal, South Africa. The rural community, described in detail elsewhere,^21,22^ has been part of the Africa Health Research Institute’s (AHRI’s) health and demographic surveillance systems for more than 20 years. The uMkhanyakude District has a high unemployment rate and relatively poor levels of service delivery.^23^ The two most important public health problems facing women of childbearing age in the uMkhanyakude District include a high HIV incidence of 3.06 seroconversion events per 100 person-years and a high maternal mortality ratio of 650 maternal deaths per 100,000 live births.^19,20^ As shown in Figure 1, the uMkhanyakude District is located in a geographic region with a sub-tropical climate characterized by wet summers and dry winters.^24^ Maximum temperatures in the uMkhanyakude District during 2012–2016 ranged from 16°C to 43°C (average 28°C). Minimum temperatures in the uMkhanyakude District during 2012–2016 ranged from 4°C to 25°C (average 17°C). Daily temperatures during the same time period ranged from 12°C to 34°C (average 22°C). A summary of trends in maximum, minimum, and daily temperatures during the period 2012–2016 in the uMkhanyakude District is provided as Figure S1. Total fertility rates have declined steeply in South Africa, likely the result of increased access to education and high-quality family planning programs which have been implemented in the country.^25^ The STROBE Guidelines were consulted when conducting this research.^26^

### Data sources

AHRI has conducted regular population-based surveys in the rural community since 2000 as part of its health and demographic surveillance system. The surveys strive to be inclusive of all households and individuals in the rural community, with an overall response rate of > 98%. These surveys also incorporate pregnancy history and pregnancy notification questionnaires to monitor child and maternal health trends in the rural community.^27^ Both the pregnancy history and pregnancy notification questionnaires have been pilot tested. With regard to the percentage of the population that the pregnancy questionnaires were pre-tested on, this specific information is unavailable as the pregnancy questionnaires were first implemented over 20 years ago and the data from the initial piloting of the questionnaires have since been lost. The pregnancy history questionnaire is administered as a baseline assessment to all women during their first survey participation (i.e. when they are first enrolled in the AHRI health and demographic system) and records details on all prior pregnancies. This includes data on the number of prior pregnancies the woman had, the number of weeks woman was pregnant for each pregnancy, the date of pregnancy outcome for each prior pregnancy, and the pregnancy outcome (live birth, stillbirth, or abortion). Pregnancy notification questionnaires (administered biannually until 2011, and triannually since 2012) collect pregnancy information on women already enrolled in the AHRI health and demographic surveillance system who are currently pregnant at the time of the survey or who report pregnancies between survey waves.^27,28^ The pregnancy notification questionnaire collects information on the date of last menses, antenatal care attendance, singleton/multiple births, the date of pregnancy outcome, and the pregnancy outcome.^27^ In rare instances, some pregnancy outcomes might not have been established as the woman and all members of her household (who might have provided information on the pregnancy outcome) migrated from the rural community. Similarly, if the pregnancy was still ongoing at the time of the last survey, then the pregnancy outcome would have been marked as “ongoing.” Data from the completed pregnancy history and pregnancy notification forms are collated in a pregnancy registry, which is updated on a regular basis and stored as an electronic database. The pregnancy registry and the questionnaires used to collect the data are available via the AHRI online data repository.^29^

Data for other variables which might influence pregnancy outcomes are collected in other demographic and health questionnaires that are also administered during the population-based survey.^21,22^ This includes sociodemographic characteristics, such as age, socioeconomic status based on a household asset index, marital status (both formal and informal unions), distance between the survey participant’s place of residence and the nearest healthcare clinic, educational level, and infectious diseases which are endemic to this region of South Africa (HIV and tuberculosis). Temperature data in the study locale for the duration of each pregnancy were obtained from the South African Weather Service.^30^

### Measures

The study outcome, miscarriage, was defined as a pregnancy which ended before 20 weeks gestation.^31^ This was established for each pregnancy by calculating the difference in weeks between the date that the pregnancy ended and the date of conception. The date of conception was determined using the date of the last menses plus 14 days. Maternal exposure to heat was measured during the month (30 days) prior to conception (T1) and the week preceding the outcome (either a miscarriage or no miscarriage, T2) for each pregnancy. The decision to use the two time points (T1 and T2) in the current study was based on these exact or similar time points being used in other published studies of temperature exposure and pregnancy outcomes.^13,15,17^ Temperature exposure throughout the duration of the pregnancy was not investigated due to an unpublished preliminary analysis (conducted by the first author) revealing a wide range of gestational duration among women who had miscarriages (range: 2.0–19.5 weeks). This would have been difficult to standardize in our planned analysis. Heat exposure was operationalized as a continuous variable and defined as the number of days that a mother was exposed to a mean daily temperature of > 26.6°C (equivalent to a mean daily temperature of > 80°F) during T1 or T2. This definition of heat exposure was adopted from the related studies on maternal exposure to heat and subsequent pregnancy outcomes.^32–34^ and better captures weather extremes and temperature variation within each time period being investigated.

### Eligibility criteria

We reviewed records from AHRI population-based surveys for all singleton pregnancies conceived between 1 January 2012 and 30 November 2016 in women aged 15–49 years. Our study was restricted to singleton pregnancies for the following reasons: multiple pregnancies (e.g. twins or triplets) only constituted a small proportion of reported pregnancies during the study period (2%); the risk of adverse pregnancy outcomes is higher in multiple pregnancies when compared with singletons,^35–37^ and thus, including multiple pregnancies could have introduced more bias into our analysis; and finally, there might be different pathways for adverse pregnancy outcomes between singleton and multiple pregnancies, entailing potential differences in risk factors (covariates for our adjusted analysis) between the two groups.^38^ We excluded pregnancies that were electively aborted, pregnancies where the woman was lost to follow-up, pregnancies in which the miscarriage outcome (miscarriage—yes or no) could not be established within 20 weeks of gestation, pregnancies where there were clear errors in the recording of dates of last menses or dates when the pregnancy ended, and pregnancies which were not reported by the mother within 1 year of being conceived (excluded due to possible recall bias around menses dates or pregnancy outcomes at the 20th week of gestation). Pregnancies which ended in stillbirth were retained in our study sample (as non-miscarriages) because these fetuses are viable up to the 20th week of gestation. The underlying mechanisms of stillbirth and miscarriage are potentially different,^39,40^ which also necessitates investigation of miscarriage on its own rather than including it in a composite outcome (i.e. pregnancy loss: miscarriage or stillbirth).

### Statistical analysis

R version 3.6.2 (R Foundation for Statistical Computing, Vienna, Austria) was used to analyze the study data.^41^ We used descriptive statistics to summarize the distribution of various pregnancy-related and maternal characteristics in our study sample. Binary logistic regression was used to investigate the relationship between maternal exposure to heat (independent variable) and miscarriage (dependent variable). We selected covariates for inclusion in our multivariate model based on our prior research.^42^ These covariates included survey year, maternal age, socioeconomic status, education level, distance between the mother’s place of residence and the nearest healthcare clinic, HIV, and tuberculosis. We conducted separate models for each time window under investigation (T1 and T2). The results of this primary analysis are presented as odds ratios (OR) with 95% confidence intervals (95% CIs). We also conducted a sensitivity analysis, wherein we accounted for whether the pregnancy was conceived during a high temperature month (December, January, February, and March). High temperature months were established by plotting mean daily temperatures for each month of the year (Figure S2). We present the results of the sensitivity analysis as OR with 95% CI. We fitted generalized estimating equations (GEE)^43^ to account for the correlation of responses within women’s repeated measures. The results of the GEE (with a logit link function, binomial distribution, exchangeable correlation structure) are presented as OR with 95% CI. We used restricted cubic spline regression to plot the relationship between maternal exposure to heat and the odds of miscarriage.

## Results

**Figure 2.**
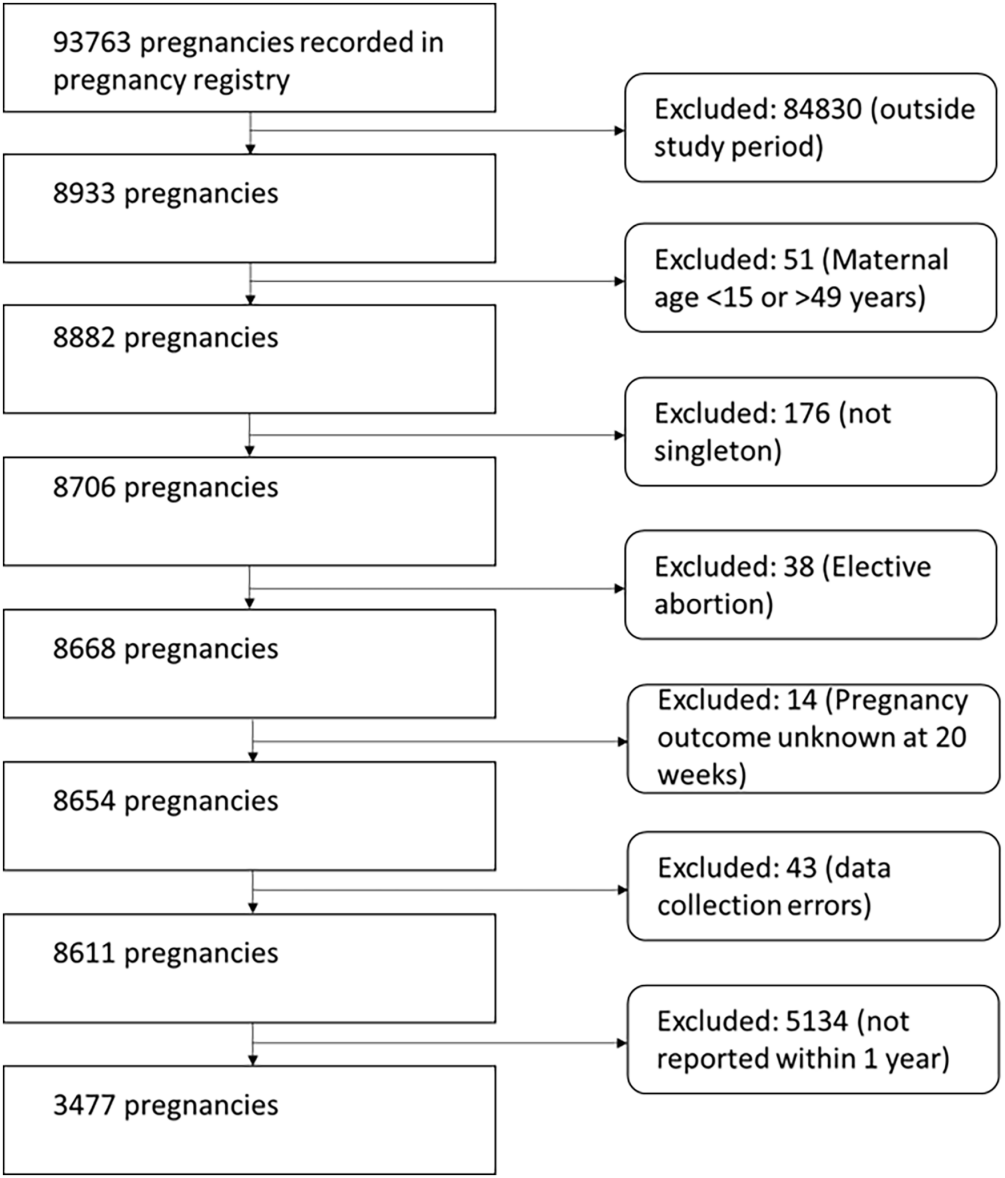
Derivation of the study sample.

As per Figure 2, our final study sample comprised 3477 pregnancies, of which 105 pregnancies ended in miscarriage (3.0%). A total of 92 out of the 105 (92%) miscarriages occurred during the first 4 weeks of gestation. Our study sample (N = 3477 pregnancies) is described in Table 1. With the exception of 2012, the remaining 4 years of the study period contributed an equal proportion of pregnancies to the analysis. Approximately one-third of pregnancies were conceived during a high temperature month. The mean number of hot days that mothers were to during T1 was 2.84 (95%CI: 2.71–2.97) days. The mean number of hot days that mothers were exposed to during T2 was 1.25 (95%CI: 1.19–1.31) days. The maternal population was young and poverty levels were high. Most of the mothers resided close to a healthcare facility. Just under half of all mothers completed high school. Nearly three-quarters of mothers were married at the time of their pregnancy. HIV and tuberculosis were confirmed as important infectious disease in women of reproductive age in our setting. Finally, the majority of women reported prior pregnancies, of which a small proportion reported a prior pregnancy ending in a miscarriage.

**Table 1.** Description of the study sample.

Characteristic	Summary statistic
Pregnancy characteristics	
Year pregnancy was conceived, n (%)	
2012	928 (26.7)
2013	683 (19.6)
2014	676 (19.4)
2015	509 (19.6)
2016	681 (19.6)
Pregnancy conceived during high temperature month?	
No, n (%)	2212 (63.6)
Yes, n (%)	1265 (36.4)
Maternal temperature exposure	
Number of hot days during the month prior to conception, T1	
Mean (95% CI)	2.84 (2.71–2.97)
Number of hot days during the week preceding the outcome (either a miscarriage or no miscarriage), T2	
Mean (95% CI)	1.25 (1.19–1.31)
Maternal characteristics	
Age at conception in years	
Mean (SD)	24.9 (6.5)
>35 years old, n (%)	270 (7.8)
Low socioeconomic status*	
No, n (%)	2376 (68.3)
Yes, n (%)	1101 (31.7)
Resides within 5 km of a healthcare clinic, n (%)	
No	368 (10.6)
Yes	3109 (89.4)
Completed high school, n (%)	
No	1910 (54.9)
Yes	1567 (45.1)
Married, n (%)	
No	983 (28.3)
Yes	2494 (71.7)
HIV serostatus, n (%)	
Unknown	2112 (60.7)
Negative	948 (27.3)
Positive	417 (12.0)
Tuberculosis, n (%)	
No	3387 (97.4)
Yes	90 (2.6)
Prior pregnancy history	
No prior pregnancies, n (%)	75 (2.2)
Prior pregnancies, no miscarriages reported, n (%)	3300 (94.9)
Prior pregnancies, miscarriages reported, n (%)	102 (2.9)

95% CI: 95% confidence interval.

* Socioeconomic status was based on asset ownership in the household to which the mother belonged. The AHRI database system automatically computes a socioeconomic index score using household survey information on house ownership, water source, energy, toilet type, electricity, and ownership of assets that can be used for consumption, production, or both. The median socioeconomic index score was used as the cut-off point to define low and high socioeconomic status—women with a socioeconomic index score below the median index score were classified as having a “low socioeconomic status.” Women with a socioeconomic index score greater than or equal to the median index score were not classified as having a “low socioeconomic status.”

**Figure 3.**
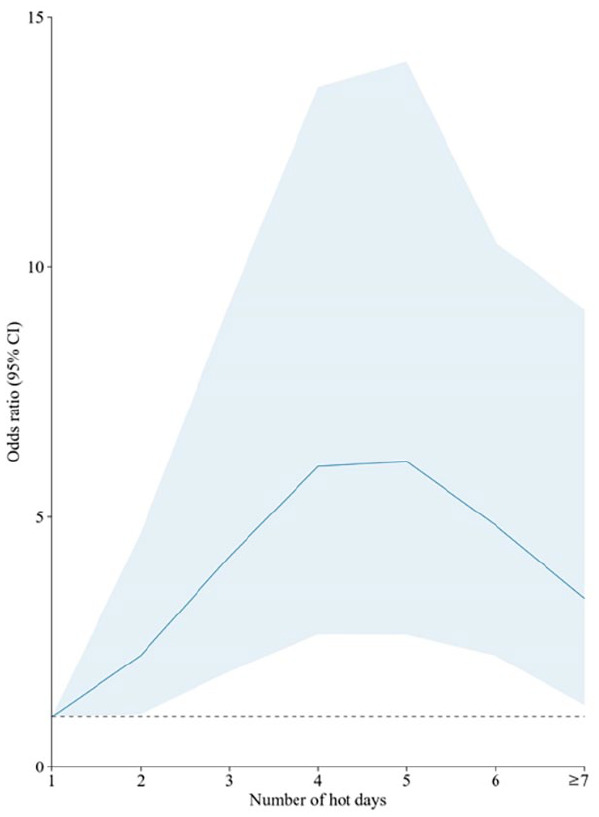
Odds of miscarriage in relation to the number of hot days that mothers were exposed to during the month preceding conception (Dark blue line, 95% CI shown as a light blue band).

Table 2 presents the results of the binary logistic regression analyses. After adjusting for potential confounders, we observed a 26% higher odds of miscarriage associated with each additional hot day during T1 (adjusted OR: 1.26; 95% CI: 1.15–1.38). No significant associations were observed between heat exposure during T2 and miscarriage (OR: 0.94, 95% CI: 0.73–1.20). Our sensitivity analysis, which accounted for whether the pregnancy was conceived during a high temperature month, confirmed the general findings from our primary regression models investigating the impact of maternal heat exposure during T1 (adjusted OR: 1.26; 95% CI: 1.15–1.38) and T2 (adjusted OR: 0.94; 95% CI: 0.73–1.22) on miscarriage risk. The results of the GEE sensitivity analysis confirmed the finding from our primary analysis, which showed an increased odds of miscarriage for each additional hot day that a mother was exposed to (adjusted OR: 1.25, 95% CI: 1.15–1.36). The relationship between the total number of hot days that mothers were exposed to during T1 and the odds of miscarriage (Figure 3) initially followed a J-shaped curve (when mothers were exposed to between 1 and 5 days of heat), and then steeply declined (when mothers were exposed to 6 or more days of heat). A possible explanation for this is that there were very few women who were exposed to 6 or more days of heat in the study sample, and thus that particular section of the graph would be less accurate.

**Table 2.** Results of the multivariate analyses investigating the relationship between maternal exposure to heat and miscarriage.

Model	OR (95% CI)
Per additional hot day that mothers were exposed to during T1*	1.26 (1.15–1.38)
Per additional hot day that mothers were exposed to during T2*	0.94 (0.73–1.20)

T1: Month preceding conception, T2: Week preceding the date of the pregnancy outcome (either a miscarriage or no miscarriage).

OR: odds ratio, 95% CI: 95% confidence interval.

* Multivariate model adjusted for maternal age, socioeconomic status, education level, marital status, distance between the mother’s place of residence and the nearest healthcare clinic, HIV, tuberculosis, mother’s prior pregnancy history, and the year in which the pregnancy was conceived. When both time periods are included in the same model, the OR for T1 was 1.27 (95% CI: 1.15–1.39) and the OR for T2 was 1.09 (95% CI: 0.82–1.41).

## Discussion

Our analysis of pregnancy data from one of the largest demographic and health surveillance platforms on the African continent demonstrates a clear relationship between maternal heat exposure during the month preceding conception and subsequent miscarriage. A large proportion of miscarriages in this study occurred within the first 4 weeks post-conception. This finding is aligned with other evidence which suggests that oocytes are particularly sensitive to high temperatures.^17^ More specifically, our adjusted statistical analysis found that the risk of miscarriage increased by 26% for each additional hot day during the month preceding conception. This relationship was illustratively confirmed when the odds of miscarriage were plotted according to the number of hot days that a mother was exposed to during the month preceding conception, revealing a J-shaped curve.

The observed harmful effect of higher temperatures during the preconception phase in our study is in keeping with the findings of other studies investigating the impact of temperature exposure during the preconception phase on adverse pregnancy outcomes from China and the United States. Guo et al.^44^ demonstrated that higher temperatures during preconception were associated with a 23% higher odds of preterm birth (OR: 1.23, 95% CI: 1.17–1.30) among pregnant women from 132 cities in China. Similarly, Ha et al.^45^ demonstrated a trend toward an increased risk of small for gestational age births and low birth weight following exposure to high temperatures during preconception in women from 12 sites in the United States (Relative Risk (RR) for small for gestational age: 1.01, 95% CI: 0.95–1.07; RR for low birth weight: 1.01, 95% CI: 0.88–1.17). Therefore, our research not only adds to the existing evidence from across the world which shows an association between heat exposure during the preconception phase and a higher risk of adverse pregnancy outcomes^11^ but also highlights the importance of increases in temperatures from climate change on pregnancy outcomes in sub-Saharan Africa.

In their systematic review, Chersich and colleagues reported an increased odds of 5% for stillbirth or preterm birth per 1°C increase in temperature exposure during pregnancy.^11^ More recently, studies from China,^12,13^ Iran,^14^ Australia,^15^ and Taiwan^16^ have confirmed the harmful association between high ambient temperature exposure and adverse pregnancy outcomes. Our study did not find an independent association between prenatal exposure to high temperatures and miscarriage. It is possible that our observed lack of association between heat exposure during the prenatal period and miscarriage might be explained by the increased sensitivity of early processes, such as oogenesis, oocyte maturation, fertilization development, and implantation rate to heat exposure.^46^ Nevertheless, when the findings of existing studies examining the prenatal period are taken into consideration with the findings of our research, it shows that women of reproductive age are vulnerable to the adverse effects of high temperatures both prior to conceiving and during their pregnancy. Chersich et al.^11^ propose several potential interventions for addressing heat exposure in women of reproductive age which may reduce their risk of adverse pregnancy outcomes. Some of the proposed interventions which might be more applicable to the preconception phase include heat reduction by improving natural ventilation in homes, making fans and cold potable water available, and conveying the risks of heat exposure to women of reproductive age.^11^ However, these proposed interventions might ultimately not be feasible in sub-Saharan Africa in the near future due to the ongoing resource constraints in this setting. Thus, there remains a need for research which seeks to establish appropriate interventions for reducing the impact of high temperatures on women’s reproductive health and pregnancy outcomes in resource-limited sub-Saharan Africa.

Our study had several strengths, the two most notable of which were our use of data from one of Africa’s largest rural population-based cohorts; and our use of consistently measured, accurate temperature data from a regional weather station. The main limitation of our study was that the pregnancy outcomes data collected during the AHRI surveys are potentially susceptible to recall bias and underreporting of miscarriages. We therefore urge caution when interpreting the findings of our study. Population-based health and demographic surveys generally lack the resources to clinically assess pregnancies, and lower than expected miscarriage rates are also reported in other large population-based health and demographic surveys from similar resource-constrained lower- and middle-income countries, such as India.^47,48^ We anticipate that in future, technological advancements would facilitate better clinical assessment of pregnancies in population-based health and demographic surveys. Other limitations of our study include that the pregnancy questionnaire was not pre-tested, we did not conduct a power analysis for our sample size, we did not have humidity measurements available to calculate Wet Bulb Globe Temperature, we could not adjust our analyses for air pollution exposure as these data were not available to us, and that there might be additional unmeasured variables which were beyond the scope of the AHRI surveys that might have introduced some residual confounding in our analysis. Finally, our use of a narrower time window (and possibly not enough time to measure the exposure) for T2 might explain why there was no significant finding for this time window. We once again urge caution when interpreting our findings.

## Conclusion

We report a clear relationship between maternal exposure to heat during the month preceding conception and miscarriage in our sub-Saharan African setting. We posit that in the absence of feasible interventions, the effects of increasing temperatures from progressive climate change will likely exacerbate the current reproductive health challenges faced by women in sub-Saharan Africa.

## Supplemental Material

sj-docx-1-whe-10.1177_17455057241259171 – Supplemental material for Maternal exposure to heat and its association with miscarriage in rural KwaZulu-Natal, South Africa: A population-based cohort study Supplemental material, sj-docx-1-whe-10.1177_17455057241259171 for Maternal exposure to heat and its association with miscarriage in rural KwaZulu-Natal, South Africa: A population-based cohort study by Yoshan Moodley, Kwabena Asare, Frank Tanser and Andrew Tomita in Women’s Health

## Declarations

**Ethics approval and consent to participate:** AHRI’s active health and demographic surveillance platform has been approved by the Biomedical Research Ethics Committee of the University of KwaZulu-Natal, South Africa (Protocol reference: BE290/16). Written consent for households (including all the adult and minor inhabitants of the households) participating in the AHRI health and demographic surveys was provided by the head of the household (an adult > 18 years of age).

**Consent for publication:** Written consent for households (including all the adult and minor inhabitants of the households) participating in the AHRI health and demographic surveys was provided by the head of the household (an adult > 18 years of age), during which permission was also given for the use and publication of the collected data for research purposes.

**Availability of data and materials:** The datasets analyzed in this study are available on AHRI’s Online Data Repository (https://data.ahri.org/index.php/home). The pregnancy questionnaire can be accessed directly through the following link: https://data.ahri.org/index.php/catalog/1091/related-materials
