# Supplementary material for: Maternal exposure to heat and its association with miscarriage in rural KwaZulu-Natal, South Africa: A population-based cohort study
Source: Womens Health (Lond). 2024 Jul 26;20:17455057241259171. doi: 10.1177/17455057241259171 (PMC11282531; doi:10.1177/17455057241259171)
Supplement: sj-docx-1-whe-10.1177_17455057241259171 – Supplemental material for Maternal exposure to heat and its association with miscarriage in rural KwaZulu-Natal, South Africa: A population-based cohort study [file sj-docx-1-whe-10.1177_17455057241259171.docx]

**Supplemental file**


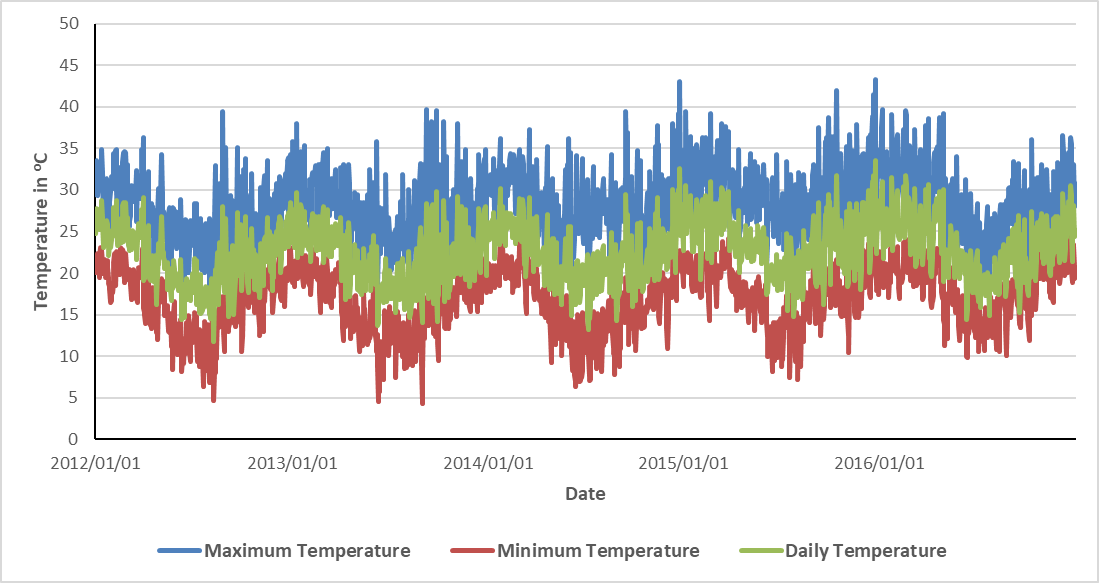


**Fig. S1.** Trends in maximum, minimum, and daily temperatures in the uMkhanyakude District of KwaZulu-Natal, South Africa (2012-2016)


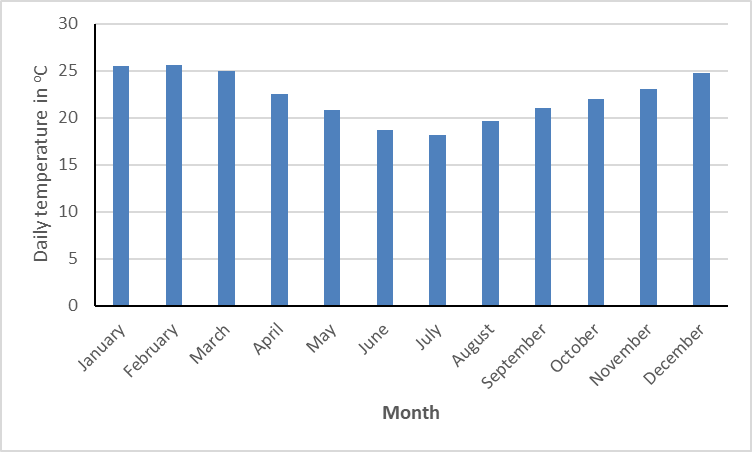


**Fig. S2.** Monthly daily temperatures in the uMkhanyakude District of KwaZulu-Natal, South Africa (2012-2016)
